# Direct Detection of Fe(II) in Extracellular Polymeric Substances (EPS) at the Mineral-Microbe Interface in Bacterial Pyrite Leaching

**DOI:** 10.1264/jsme2.ME15137

**Published:** 2016-03-05

**Authors:** Satoshi Mitsunobu, Ming Zhu, Yasuo Takeichi, Takuji Ohigashi, Hiroki Suga, Muneaki Jinno, Hiroko Makita, Masahiro Sakata, Kanta Ono, Kazuhiko Mase, Yoshio Takahashi

**Affiliations:** 1 Department of Environmental Conservation, Graduate School of Agriculture, Ehime UniversityTarumi, Matsuyama 790–8566Japan; 2 Graduate Division of Nutritional and Environmental Sciences, University of ShizuokaYada, Suruga-ku, Shizuoka 422–8526Japan; 3 Institute of Materials Structure Science, High-Energy Accelerator Research Organization (KEK)Oho, Tsukuba, Ibaraki 305–0801Japan; 4 The Graduate University for Advanced Studies1–1 Oho, Tsukuba 305–0801Japan; 5 UVSOR facility, Institute for Molecular ScienceMyodaiji, Okazaki 444–8585Japan; 6 Department of Earth and Planetary Systems Science, Hiroshima UniversityKagamiyama, Higashi-Hiroshima, Hiroshima 739–8526; 7 Toyama Co. Ltd.4–13–16 Hibarigaoka, Zama, Kanagawa 252–0002Japan; 8 Japan Agency for Marine-Earth Science and Technology (JAMSTEC)Natsushima-cho, Yokosuka, Kanagawa 237–0061Japan; 9 Department of Earth and Planetary Science, The University of TokyoHongo, Bunkyo-ku, Tokyo 113–0033Japan

**Keywords:** Bacterial mineral leaching, pyrite, chemical speciation, X-ray microscopy

## Abstract

We herein investigated the mechanisms underlying the contact leaching process in pyrite bioleaching by *Acidithiobacillus ferrooxidans* using scanning transmission X-ray microscopy (STXM)-based C and Fe near edge X-ray absorption fine structure (NEXAFS) analyses. The C NEXAFS analysis directly showed that attached *A. ferrooxidans* produces polysaccharide-abundant extracellular polymeric substances (EPS) at the cell-pyrite interface. Furthermore, by combining the C and Fe NEXAFS results, we detected significant amounts of Fe(II), in addition to Fe(III), in the interfacial EPS at the cell-pyrite interface. A probable explanation for the Fe(II) in detected EPS is the leaching of Fe(II) from the pyrite. The detection of Fe(II) also indicates that Fe(III) resulting from pyrite oxidation may effectively function as an oxidizing agent for pyrite at the cell-pyrite interface. Thus, our results imply that a key role of Fe(III) in EPS, in addition to its previously described role in the electrostatic attachment of the cell to pyrite, is enhancing pyrite dissolution.

Bioleaching is a mineral leaching process performed by various biological metabolic pathways (29, 30). The microbial bioleaching of metal sulfides has been used as a low-cost engineering process to extract metals from sulfidic ores, particularly low-grade ores, including pyrite (FeS_2_) and chalcopyrite (CuFeS_2_) (22, 29, 30). The microbial bioleaching of metal sulfide also contributes to the formation of environmentally detrimental acid mine drainage (AMD), the acidic nature and heavy-metal constituents of which may seriously contaminate soil as well as surface- and groundwater in various parts of the world. Thus, in order to improve industrial bioleaching and reduce the formation of AMD, a better understanding of the mechanisms involved is of crucial importance. The rate of bioleaching of metal sulfides is faster than that of chemical leaching (19, 22). In the case of pyrite bioleaching, the biotic leaching rate was previously reported to be 30–40-fold faster than that of abiotic leaching (19), which suggests that microbes use special processes to efficiently leach minerals.

Extensive efforts have been made to identify the mechanisms underlying bioleaching. It is now generally accepted that there are two modes of bioleaching of metal sulfides: “contact” and “non-contact” leaching (20, 23, 30). In the case of pyrite leaching, non-contact leaching is mainly promoted by planktonic microbes, which oxidize the reduced Fe and S in solution. The resulting Fe(III) ions come into contact with a mineral surface and work as an oxidizing agent, which promotes mineral dissolution. Contact leaching is a process in which microbial cells attach to the mineral surface and promote mineral dissolution locally at the microbe-mineral interface. Previous studies showed that the extracellular polymeric substances (EPS) produced by leaching microbes are a key agent in the enhancement of mineral dissolution in contact leaching (6, 17). However, little is known of the role of microbial EPS in contact bioleaching, at the scale of 100 nanometers (1), because of difficulties with the direct chemical speciation of metals and biomolecules in EPS due to high spatial resolution in analyses. Although contact leaching has so far been mainly studied using indirect techniques, direct observations of the chemical speciation of metals and biomolecules is necessary for a better understanding of the mechanisms underlying contact leaching and bioleaching in general.

In the present study, we investigated the mechanisms underlying the contact leaching process in pyrite bioleaching using a direct chemical speciation technique, scanning transmission X-ray microscopy (STXM)-based near-edge X-ray absorption fine structure (NEXAFS) analysis. The STXM-based NEXAFS technique is a new powerful tool for researching microbe-mineral interactions. To date, there have been very few applications of the STXM technique to the study of bioleaching (16). The application of a STXM-based NEXAFS analysis, having high spatial resolution (less than 50 nm) and allowing the direct detection of chemical species at high elemental specificities, will enable us to directly examine microbe-pyrite interface interactions.

## Materials and Methods

### Bacterial strain and cultivation

The acidophilic chemolithoautotrophic bacterium, *Acidithiobacillus ferrooxidans*, was used as the leaching microbe in the present study. *A. ferrooxidans* has been widely used in studies on industrial bioleaching and the formation of AMD (9, 22), and strain JCM7812 was used in this study. This strain, which was originally isolated from a sulfur and Fe sulfide mine in Japan, uses energy from the oxidation of reduced Fe and S for growth and fixes CO_2_ and N_2_ from the atmosphere (32). The strain was cultivated in mineral salt medium (Mackintosh medium) with Fe(II) sulfate immediately prior to pyrite bioleaching (12).

### Pyrite bioleaching with *A. ferrooxidans*

Pyrite bioleaching was performed in a batch process using pyrite powder. Natural pyrite from the Navajún mine, La Rioja, Spain was used (3). Cubic pyrite was ground and sieved to a particle size of 50–100 μm. Powdered pyrite was subsequently cleaned with boiled 6 M HCl, rinsed with deionized water and acetone to remove Fe and S compounds, and dried at 120°C in an oven according to a previously developed procedure (24).

In pyrite bioleaching, *A. ferrooxidans* pre-cultured with Fe(II) sulfate was inoculated into 10 mL of Mackintosh medium with 5 wt% pyrite (initial total cell number: 10^8^ cells mL^−1^). Cells were aerobically incubated at 30°C in a reciprocal shaker in the dark, as described by Mackintosh (12). The initial pH of the medium was adjusted to pH 3.0 with 0.1 M H_2_SO_4_. The duration of the incubation was 35 d (5 weeks).

In order to determine the concentrations of total Fe, Fe(II), total S, sulfidic-S, and sulfate, the suspension samples were filtered through a 0.2-μm PTFE filter (Advantec), and the filtrate was analyzed. Total Fe and S concentrations were determined by ICP-OES (Varian, 730-ES), and aqueous Fe(II) and sulfide concentrations by the phenanthroline technique and methylene-blue technique, respectively, using a spectrophotometer (27). The abundance of sulfate was measured by ion chromatography (Thermo Fisher Sci., ICS-5000plus).

The concentrations of cells in suspension were determined by fluorescent staining as previously described (7, 9). The samples used in cell counting were not applied to the STXM analysis. A 1-mL aliquot of the suspension was vacuum-filtered onto a black polycarbonate membrane filter (pore size 0.2 μm, Advantec). The cells collected were stained with Syto9 (Life Technologies) at a final concentration of 0.1 mM for 10 min. Imaging and counting were performed by epifluorescence microscopy (Olympus, BX-51) with a Canon X5 CCD camera. As described in a previous study (7), cells in 15–20 randomly distributed fields on the membrane filter were counted.

### STXM-based C 1s, Fe 2p NEXAFS, and Fe K-edge XANES analyses

STXM analyses for C 1s and Fe 2p NEXAFS were conducted at two STXM apparatuses installed in BL-13A at KEK-PF (Tsukuba) (26) and BL-4U at UVSOR (Okazaki) (18) in Japan. The theoretical spatial and spectral resolutions of both STXM apparatuses were less than 50 nm and ±0.1 eV, respectively. STXM analyses were performed at room temperature (RT) and ~1/6 atm He. Regarding sample preparation, 1 mL of suspension was transferred to a sterile plastic tube and washed gently three times with Fe-free Mackintosh medium (pH 3.0) to remove excess salts. A small amount of the suspension was dropped onto a Si_3_N_4_ membrane (Silson, thickness 100 nm) and air-dried slowly at RT (10). All STXM data processing was carried out using the IDL package aXis2000 software (Hitchcook, an IDL-based analytical package, http://unicorn.mcmaster.ca/aXis2000.html). A careful examination showed that there was no apparent photo alteration of C or Fe during the STXM analyses performed in the present study.

Iron K-edge X-ray absorption near edge structure (XANES) spectra were also measured in order to obtain information for bulk Fe species in solid samples. The analysis was performed at the beamlines BL01B1 at SPring-8 (Hyogo, Japan) and BL4A at KEK-PF (Tsukuba, Japan) with a Si(111) double-crystal monochromator and two mirrors. In the XANES analysis, a solid sample was collected by vacuum-filtration on a PTFE filter with a pore size of 0.2 μm and freeze-dried. The solid collected was diluted to *ca.* 1 wt% with boron nitride and pelleted for the transmission detection mode for Fe XANES. The energy calibration for Fe XANES was performed using a pre-edge peak maximum of hematite at 7.113 keV.

All XANES data processing was performed using the XAFS analysis package, REX2000 (Rigaku). All model compounds for C NEXAFS such as albumin, sodium alginate, agarose, *Escherichia coli* DNA, and 1,2-dipalmitoyl-sn-glycero-3-phosphoethanolamine were obtained from a Japanese reagent provider, Wako Pure Chemicals. Natural biotite and siderite were obtained from the Japanese mineral provider, Hori Mineralogy as Fe NEXAFS and XANES model compounds. The secondary Fe minerals, ferrihydrite, goethite, and hematite were synthesized as described by Mitsunobu *et al.* (15). In addition, Fe(II) and Fe(III) complexed with organic ligands were synthesized as described by Mitsunobu *et al.* (15) and Chan *et al.* (4). Alginate was selected as a well-characterized acidic polysaccharide. Fe(II) (or Fe[III]) was added to sodium alginate as dissolved Fe(II) (or Fe[III]) chloride, in which Fe:COO^−^ was adjusted to ~1:100. A small amount of the Fe-alginate suspension was dropped onto the Si_3_N_4_ membrane and air-dried at RT prior to STXM measurements.

### SEM analysis

The morphology of the pyrite surface was examined using scanning electron microscopy (SEM; Keyence VE-9800). A 1-mL suspension was taken and transferred to a 2-mL sterile plastic tube. The supernatant solution was decanted, and an ethanol dehydration series of 50%, 70%, 90%, and 100% (v/v) was then performed immediately prior to the analysis. The sample was mounted on carbon tape, dried, and analyzed after osmium coating.

### Staining of extracellular polysaccharides with lectin

The staining of extracellular polysaccharides of *A. ferrooxidans* was performed using a lectin technique as previously described by Mangold *et al.* (13). The technique has been used for the selective staining of EPS of *A. ferrooxidans*. A 1-mL suspension was transferred to a sterile plastic tube and washed gently twice with 1 mL of deionized water. Nucleic acids were then stained with Syto9 at a final concentration of 0.1 mM for 10 min. After one wash, EPS were stained for 30 min with Cy3-labeled Concanavalin A (ConA; Protein Mods) at a final concentration of 50 μg mL^−1^. After two washes, the stained suspension was mounted on a glass slide or polycarbonate membrane filter. Imaging was performed by epifluorescence microscopy.

## Results and Discussion

### Solution chemistry and SEM analysis

**Fig. 1:**
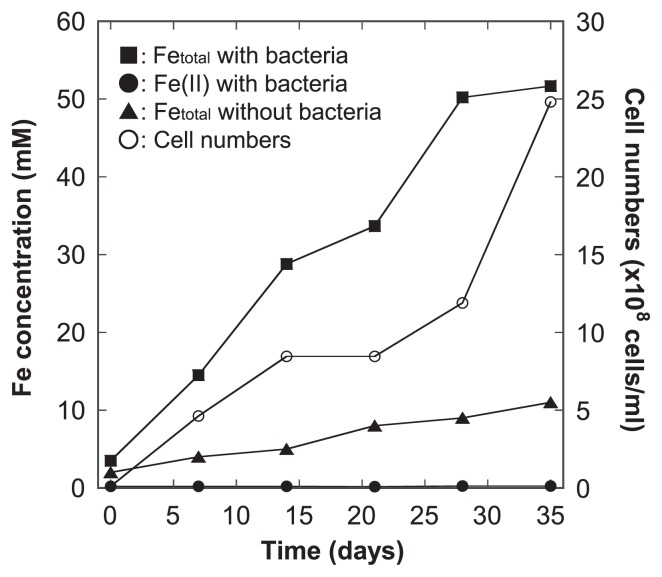
Concentrations of total Fe and Fe(II) with/without bacteria and cell numbers. The initial amount of pyrite was 0.5 g in a total volume of 10 mL at an initial pH 3.

Fig. 1 shows changes in aqueous Fe concentrations and cell numbers as a function of time with and without *A. ferrooxidans*. Total Fe concentrations and cell numbers both steadily increased throughout the incubation period. Fe concentrations were markedly higher in the presence of *A. ferrooxidans* rather than in its absence. The Fe(II) fraction was very low and less than 1% of the total Fe concentration throughout the incubation period. The positive correlation between cell numbers and aqueous Fe concentrations (*r*^2^=0.76) indicated that pyrite supported the growth of *A. ferrooxidans* and metabolic activity enhanced the leaching of pyrite. Although we also carried out measurements of sulfate concentrations (data not shown), no sulfidic S species, aqueous H_2_S, HS^−^, or S^2−^ ions, were detected during the incubation. Sulfate concentrations also increased with the incubation time, and the S/Fe molar ratio at each time point was approximately 2 (S/Fe=1.7~2.1, data not shown). pH gradually decreased over time due to the oxidation of pyrite and subsequent formation of sulfate acid, which was at approximately pH 2 at the end of the incubation. These results suggest that most of the released Fe and S were left in the aqueous phase without significant mineral precipitation because the S/Fe ratio in pyrite was theoretically 2.

**Fig. 2:**
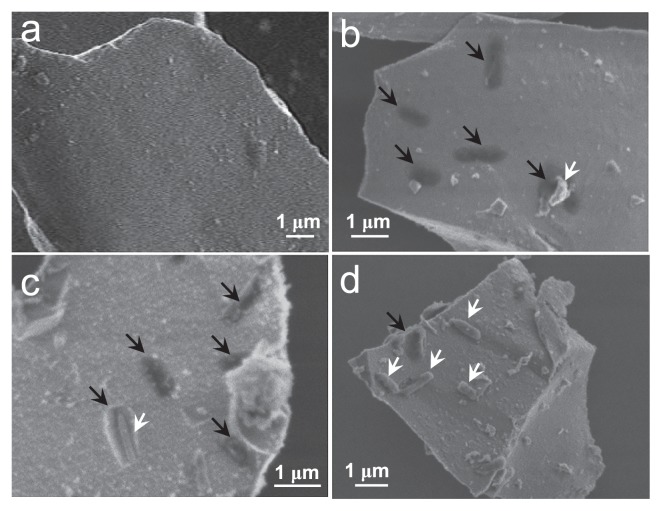
SEM images of pyrite particles after a 4-week incubation without (a) and with *A. ferrooxidans* (b)–(d). Black and white arrows in images (b)–(d) stand for rod-shaped pits and *A. ferrooxidans* cells on the pyrite surface, respectively.

The surface morphology of pyrite incubated with/without bacteria for 4 weeks was investigated by SEM (Fig. 2). A large number of rod-shaped pits (black arrows in Fig. 2b, c, and d) were observed on the surface of pyrite incubated with the bacteria (white arrows), while no pits were observed on pyrite incubated without the bacteria, as shown in Fig. 2a. The sizes of these pits were similar to the size of *A. ferrooxidans* cells (Fig. 2b, c, and d). Previous studies have demonstrated that cell-sized pits are formed during the bioleaching of pyrite due to the contact leaching process, *i.e.*, bioleaching by sessile microbes (11, 33). Thus, the appearance of pits on the surface of pyrite suggests that contact leaching occurred in the present pyrite bioleaching.

### STXM-based direct speciation of major biomolecules at the cell-pyrite interface

SEM observations suggested the occurrence of contact leaching in this experiment. In order to investigate the mechanisms underlying the contact bioleaching process, we applied STXM-based C and Fe NEXAFS to pyrite samples after the bioleaching assay. By using the STXM-based NEXAFS technique, we were able to obtain direct information on the chemical species of C and Fe at the microbe-pyrite interface.

**Fig. 3:**
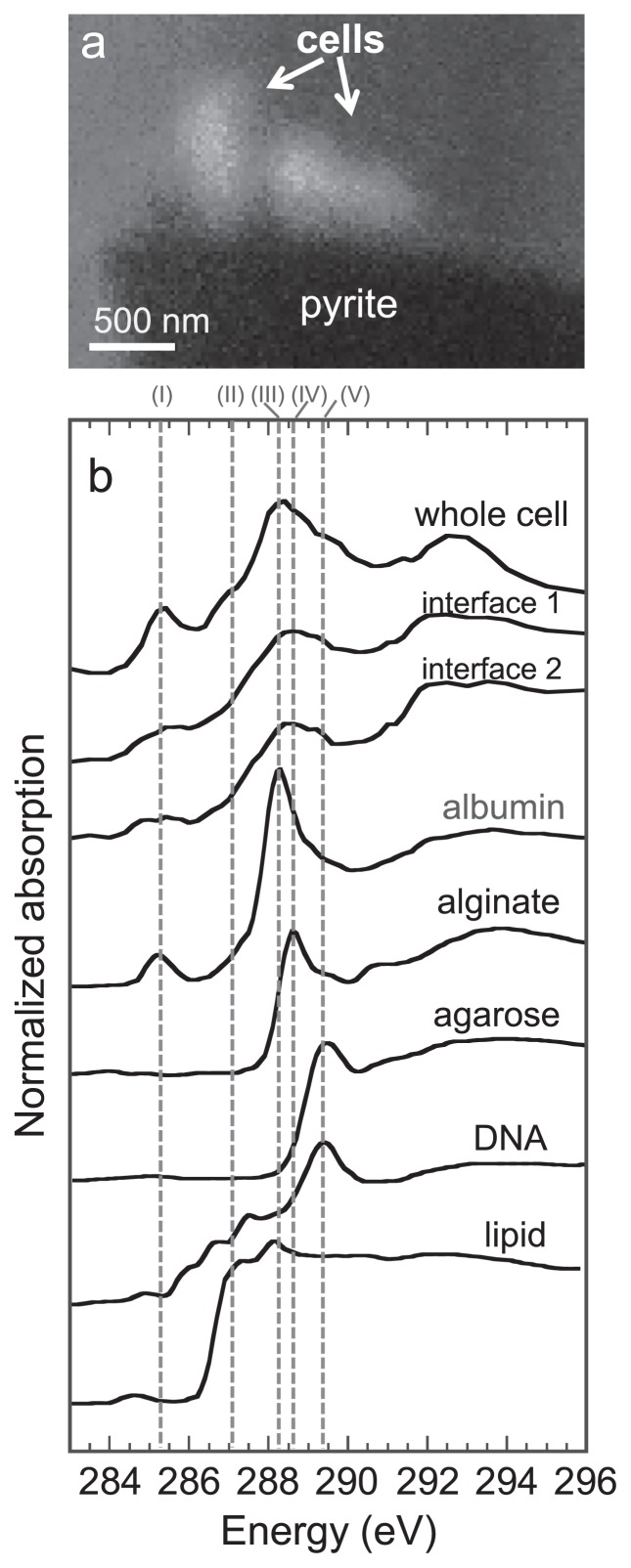
(a) STXM-based C image of sessile *A. ferrooxidans* cells on pyrite in the 2-week incubation. The image was obtained by dividing the two images at 282 eV (C 1s pre-edge energy) and 304 eV (post-edge energy). (b) STXM-based C 1s NEXAFS spectra of model compounds and sessile *A. ferrooxidans* cells. In C NEXAFS, sodium alginate (acidic polysaccharide), agarose (neutral polysaccharide), albumin (protein), *Escherichia coli* DNA (nucleic acid), and 1,2-dipalmitoyl-sn-glycero-3-phosphoethanolamine (lipid) were selected as model compounds for major microbial biomolecules, as in previous studies (5, 8). The dotted lines (I) to (V) in Fig. 3b stand for the peak energies of the major organic functional groups present in the model compounds shown in Table 1.

**Fig. 4:**
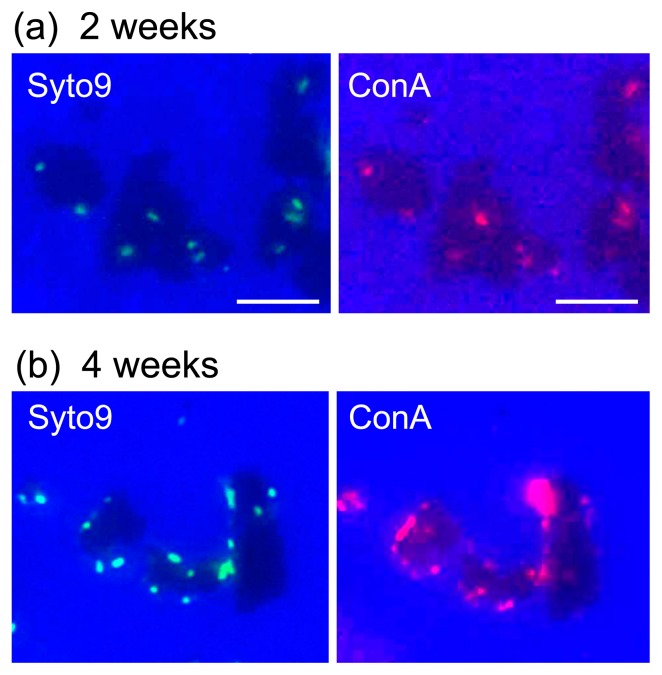
Sessile *A. ferrooxidans* cells on pyrite particles stained with Syto9 for nucleic acids, and with ConA for extracellular polysaccharides after incubations for 2 weeks (a) and 4 weeks (b). Black grains in the images show pyrite particles. All scale bars: 10 μm.

The STXM-based C images and C 1s NEXAFS spectra of *A. ferrooxidans* that attached to the pyrite particles (samples after a 2- and 4-week incubation) are shown in Fig. 3. The lower part of the C image in the 2-week sample shows a pyrite particle because the X-ray absorption of this area was saturated with an optical density of more than 2 (Fig. 3a). The spectral features of the model compounds in C NEXAFS showed clear differences among compounds representing polysaccharides, lipids, proteins, and nucleic acids (the functional group and transition corresponding to each peak are summarized in Table 1); therefore, we were able to distinguish major bacterial biomolecules using C NEXAFS. The spectrum of the whole cell area after a 2-week incubation consisted of peaks representing aromatic (I), aliphatic (II), amide (III), carboxyl (IV), and O-alkyl (V) C (whole cell in Fig. 3b). The spectral features of the whole cell were basically similar to those of a mixture of albumin, alginate, and lipids, exhibiting major peaks for aromatic at 285.2 eV (I), aliphatic at 287.3 eV (II), amide at 288.2 eV (III), and carboxyl at 288.6 eV (IV). In contrast, a significant difference was found in the C NEXAFS spectra of the cell-pyrite interface (Fig. 3b). The spectra of the interface (interfaces 1 and 2 in Fig. 3b) mainly included carboxyl (peak [IV]) and O-alkyl C (peak [V]), typical of acidic and neutral polysaccharides, whereas aromatic (I), aliphatic (II), and amide (III) peaks were smaller than those of the whole cell. A similar localization of polysaccharides was also observed in our preliminary study (16). In addition, the attached *A. ferrooxidans* cells after the 2- and 4-week incubations were successfully stained by the lectin specific for polysaccharides (Fig. 4a and b), indicating that the *A. ferrooxidans* cells that attached to pyrite were coated with polysaccharide-rich EPS (2). Hence, the spectroscopic results from C NEXAFS were consistent with those of the lectin staining analysis. Thus, these results consistently suggest that *A. ferrooxidans* abundantly produces EPS in the polysaccharides on the pyrite surface.

Previous studies based on the characterization of extracted EPS found that the chemical constituents of EPS produced by *A. ferrooxidans* varied depending on the type of growth substrate (*e.g.* pyrite and elemental S). The EPS of *A. ferrooxidans* grown on pyrite contain markedly more uronic acid residues and neutral sugars, but fewer fatty acids than EPS from cells grown on elemental S (6, 31). This is consistent with our results showing the high composition of acidic polysaccharides in EPS on the pyrite surface.

### STXM-based direct speciation of Fe at the cell-pyrite interface

**Fig. 5:**
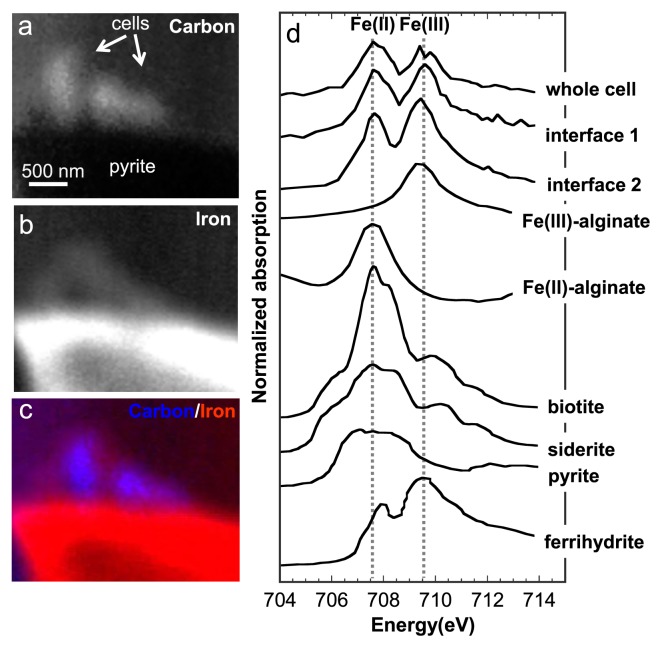
STXM-based C, Fe, and merged images (a, b, c) and Fe 2p NEXAFS spectra (d) of model compounds and sessile *A. ferrooxidans* cells on pyrite after a 2-week incubation. Pyrite, ferrihydrite, siderite, biotite, and Fe(II) and Fe(III) complexed with acidic polysaccharides (sodium alginate) were selected as the model compounds for Fe NEXAFS. Vertical lines in Fig. 5d were displayed to show the specific Fe(II) and Fe(III) peaks at 707.5 and 709.1 eV, respectively. The Fe image was obtained by dividing two images at 700 eV (Fe 2p pre-edge energy) and 730 eV (post-edge energy).

We investigated the Fe species in EPS produced at the cell-pyrite interface by the Fe NEXAFS analysis. Fig. 5 shows STXM-based Fe and C images and Fe NEXAFS of *A. ferrooxidans* attached to pyrite particles in the 2-week incubation of samples. The image in Fig. 5c shows that Fe was localized around the surface of the *A. ferrooxidans* cells. C NEXAFS demonstrated the appearance of a polysaccharide-rich layer at the cell-pyrite interface, suggesting that Fe had accumulated in this polysaccharide layer. The Fe NEXAFS spectra of samples and model compounds are shown in Fig. 5d. The absorption edge of Fe(II) species shifted to a lower energy than that of Fe(III) species, as described in a previous study (28). The vertical lines in Fig. 5d show the edge peaks characteristic for Fe(II) (707.5 eV) and Fe(III) (709.1 eV), respectively, indicating that, as reported in previous studies (25, 28), the abundances of Fe(II) and Fe(III) may be estimated using these specific peaks. The spectra of the whole cell and cell-pyrite interface both consisted of Fe(II) and Fe(III) peaks, and no clear difference was found between these spectra. Thus, the Fe species in the cell and interface were both uniformly Fe(II)/Fe(III) mixed species. The Fe species found in the cell grown on pyrite may have resulted from (i) Fe in aqueous solution, (ii) Fe complexed with ligands, and (iii) Fe in precipitates having a smaller particle size than the spatial resolution of STXM (50 nm). Regarding (i), we may exclude dissolved Fe in the leaching solution because most Fe in the leaching solution was Fe(III) and the Fe(II) fraction was less than 1%, as shown in Fig. 1. We may also rule out dissolved Fe in the bacterial cell cytoplasm because the concentration needs to approach 0.1 mM in order to be detected by STXM-NEXAFS (28). The Fe concentration in the bacterial cell cytoplasm is generally less than 0.5 μM (14). Regarding (iii), we may also rule out nanoparticulate precipitates on the basis of the Fe NEXAFS spectra because the spectral features of Fe(II) and Fe(III) in the sample did not match those of Fe(II)-bearing sulfide, carbonate, or silicate minerals (Fig. 5d). In contrast, the spectral features of Fe in the sample were similar to those of Fe(II) and Fe(III) complexed by acidic polysaccharides in model compounds. In addition, the Fe image showed the accumulation of Fe in EPS at the pyrite surface (Fig. 5c). These results are consistent with a chemical association between Fe and C through the complexation of Fe by organic functional groups involved in EPS, case (ii). Previous studies also suggested based on stoichiometric estimations that Fe is complexed with organic ligands in EPS (6, 30), which is consistent with our results.

**Fig. 6:**
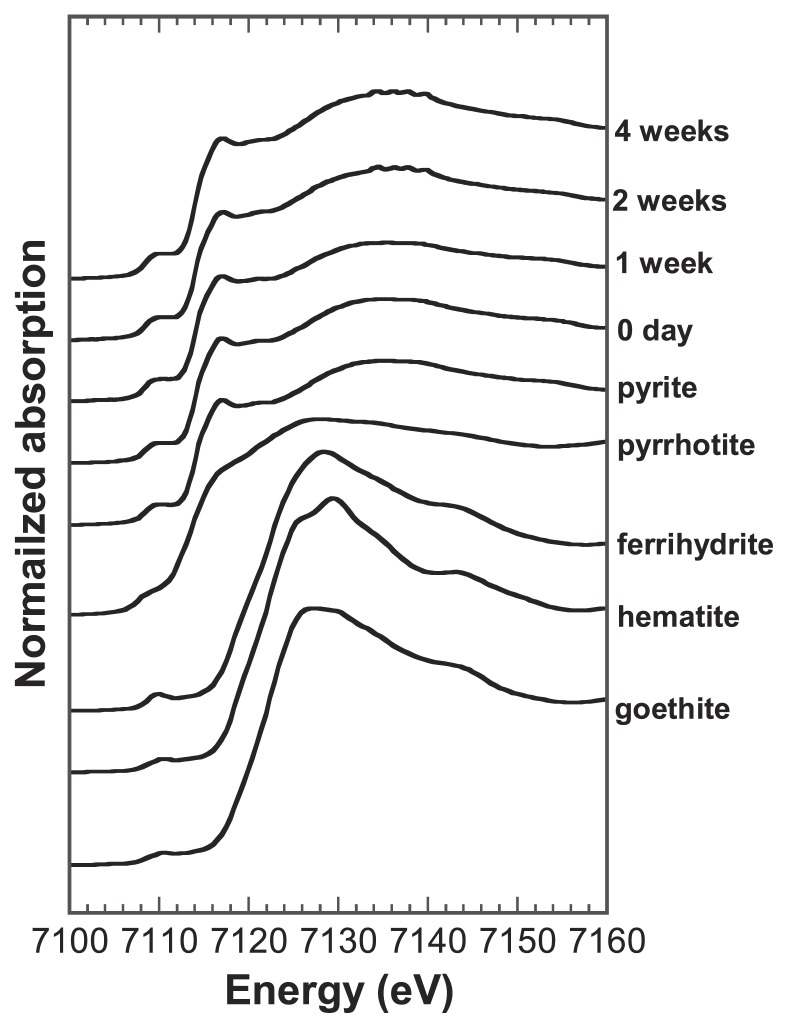
Iron K-edge XANES spectra of bulk solid samples after incubations and Fe model compounds of pyrite, pyrrhotite, ferrihydrite, hematite, and goethite.

The bulk Fe K-edge XANES analysis with quantitative fitting showed that the bulk Fe species in the solids was mainly pyrite over the incubation period and prominent amounts of other Fe species were not found (Fig. 6), indicating that the Fe(II)/Fe(III) mixed species was the Fe species observed locally at the cell-pyrite interface.

### Implication of Fe(II) detection in the interfacial EPS for the bioleaching mechanism

Nanoscale C and Fe NEXAFS analyses showed that the interfacial EPS contain significant amounts of Fe(II) in addition to Fe(III) in the sessile cell on pyrite. A plausible candidate for the Fe(II) and Fe(III) species was the complex with organic ligands in EPS. A noteworthy result was the detection of Fe(II) in the interfacial EPS. To the best of our knowledge, this is the first detection of Fe(II) in the EPS produced by bioleaching microbes.

A probable explanation for the Fe(II) detected is the leaching of Fe(II) from pyrite. The EPS of pyrite-grown *A. ferrooxidans* contain acidic polysaccharides with a carboxylic group, such as uronic acid residues (6). Organic ligands such as carboxylic groups generally increase metal solubility by complexing the metal (30), and the complexed Fe(II) has a higher bioavailability than solid Fe(II). A second possibility for the Fe(II) detected is the formation of Fe(II) by the oxidation of sulfides at the cell-pyrite interface. As stated above, *A. ferrooxidans* has the ability to change the chemical constituents in EPS, depending on the type of substrate to improve cell attachment to the substrate (6). The EPS of *A. ferrooxidans* grown on pyrite contained more uronic acid residues and complexed Fe(III) (12) than that of *A. ferrooxidans* growing on elemental S and in Fe(II) sulfate solution. The complexation of uronic acid with Fe(III) allows cells to have a net positive charge (6, 22), which means that they may preferentially attach to negatively charged pyrite by electrostatic interactions, thereby promoting the dissolution of pyrite. Researchers have also suggested another role for Fe(III) in EPS: an oxidizing agent for sulfides (21, 22); *i.e.*, the Fe(III) in EPS may abiotically oxidize sulfides in pyrite to thiosulfate and sulfate according to equations 1 and 2.

(1) $${\text{FeS}}_{2}+6{\text{Fe}}^{3+}+3{\text{H}}_{2}\text{O}\to {\text{S}}_{2}{{\text{O}}_{3}}^{2-}+7{\text{Fe}}^{2+}+6{\text{H}}^{+}$$

(2) $${\text{S}}_{2}{{\text{O}}_{3}}^{2-}+8{\text{Fe}}^{3+}+5{\text{H}}_{2}\text{O}\to 2{{\text{SO}}_{4}}^{2-}+8{\text{Fe}}^{2+}+10{\text{H}}^{+}$$

However, this interfacial process is merely speculative because no Fe(II) in EPS subsequently formed by this oxidation process has been observed to date. Previous studies determined the Fe species in EPS by indirect methods including EPS extraction and digestion, which may cause some unwanted oxidation of the Fe species during the analysis. The Fe(II) detected by our direct speciation technique, STXM-based NEXAFS, is a possible indication supporting the oxidative attack by Fe(III) in EPS, while knowledge of the nature of the S species in interfacial EPS is necessary in order to allow a more detailed discussion of this process.

In summary, this study investigated the nanoscale spatial distribution of major biomolecules such as proteins, polysaccharides, lipids, and nucleic acids as well as Fe species at the cell-pyrite interface during pyrite bioleaching by *A. ferrooxidans* using STXM-based C and Fe NEXAFS analyses. The C NEXAFS analyses directly showed that a polysaccharide-rich layer was localized at the cell-pyrite interface, indicating the production of polysaccharide-rich EPS by sessile *A. ferrooxidans*. Furthermore, combined with the Fe NEXAFS analysis, we detected Fe(II) in the interfacial EPS at the cell-pyrite interface. The detection of Fe(II) is a possible indication showing that Fe(III) in EPS functions as an oxidizing agent for the sulfides in pyrite at the cell-pyrite interface. Our results indicate that Fe(III) in EPS, as well as its previously reported role in the electrostatic attachment of cells to pyrite, is a key component enhancing pyrite dissolution. Thus, our results provide important information for understanding the mechanisms underlying bacterial mineral leaching and the formation of AMD.

## Figures and Tables

**Table 1:** Major organic functional groups present in model compounds and samples (summarized data from Chan *et al.* (5) and Keiluweit *et al.* (8))

peak	Energy (eV)	Transition	Functional group
(I)	285.2	1s→π*_C=C_	aromatic C
(II)	287.3	1s→3p/σ*	aliphatic C
(III)	288.2	1s→π*_C=O_	amide C (peptide bond)
(IV)	288.6	1s→π*_C=C_	carboxyl C (acidic polysaccharide)
(V)	289.3	1s→3p/σ*	O-alkyl C
